# Mania-related effects on structural brain changes in bipolar disorder – a narrative review of the evidence

**DOI:** 10.1038/s41380-023-02073-4

**Published:** 2023-05-05

**Authors:** Christoph Abé, Benny Liberg, Anna Luisa Klahn, Predrag Petrovic, Mikael Landén

**Affiliations:** 1 Department of Clinical Neuroscience, Karolinska Institutet, Stockholm, Sweden; 2 Quantify Research, Stockholm, Sweden; 3 Department of Psychiatry and Neurochemistry, Institute of Neuroscience and Physiology, Sahlgrenska Academy, University of Gothenburg, Gothenburg, Sweden; 4 Department of Chemistry and Molecular Biology, University of Gothenburg, Gothenburg, Sweden; 5 Center for Cognitive and Computational Neuropsychiatry, Karolinska Institutet, Stockholm, Sweden; 6 Center for Psychiatry Research, Karolinska Institutet, Stockholm, Sweden; 7 Department of Medical Epidemiology and Biostatistics, Karolinska Institutet, Stockholm, Sweden

**Keywords:** Prognostic markers, Neuroscience, Bipolar disorder

## Abstract

Cross-sectional neuroimaging studies show that bipolar disorder is associated with structural brain abnormalities, predominantly observed in prefrontal and temporal cortex, cingulate gyrus, and subcortical regions. However, longitudinal studies are needed to elucidate whether these abnormalities presage disease onset or are consequences of disease processes, and to identify potential contributing factors. Here, we narratively review and summarize longitudinal structural magnetic resonance imaging studies that relate imaging outcomes to manic episodes. First, we conclude that longitudinal brain imaging studies suggest an association of bipolar disorder with aberrant brain changes, including both deviant decreases and increases in morphometric measures. Second, we conclude that manic episodes have been related to accelerated cortical volume and thickness decreases, with the most consistent findings occurring in prefrontal brain areas. Importantly, evidence also suggests that in contrast to healthy controls, who in general show age-related cortical decline, brain metrics remain stable or increase during euthymic periods in bipolar disorder patients, potentially reflecting structural recovering mechanisms. The findings stress the importance of preventing manic episodes. We further propose a model of prefrontal cortical trajectories in relation to the occurrence of manic episodes. Finally, we discuss potential mechanisms at play, remaining limitations, and future directions.

## Introduction

Cross-sectional neuroimaging studies show that bipolar disorder is associated with structural brain abnormalities, predominantly observed in prefrontal and temporal cortex, cingulate gyrus, and subcortical regions [1–4], and less consistently in insula and visual cortex [1–8]. Large-scale studies from the ENIGMA (Enhancing Neuro Imaging Genetics through Meta Analysis) bipolar disorder working group found the most pronounced cortical alterations in pars opercularis and rostral middle frontal and fusiform cortex [2]. Subcortical abnormalities have been observed in amygdala, hippocampus, and thalamus in bipolar disorder patients. Finally, enlarged ventricles have been observed in bipolar disorder [1].

The causes of these findings remain unknown and the question whether the observed brain aberrancies presage disease onset or are consequences of disease processes cannot be resolved due to inherent limitations of cross-sectional study designs. Some observations suggest that brain abnormalities might be related to worsening along the course of illness and decline in general functioning, at least in some patients with bipolar disorder [9–13]. Such progressive deterioration of illness has—along with hypothesized neuroanatomical changes over time—been denoted ‘neuroprogression’ [13] and has also been described in bipolar disorder [12–14]. It is still disputed, however, whether bipolar disorder entails such neuroprogression [15], mainly because longitudinal studies investigating brain changes over time are scarce.

Recently, however, a few longitudinal studies of brain morphology that enable conclusions about possible brain changes in bipolar disorder have been performed. These do in fact indicate that aberrant brain changes occur. Single-center studies [16–20], multi-center studies [21], and recent reviews [13, 14, 22] have observed structural changes mainly in the prefrontal and temporal cortices. Notably, however, the largest longitudinal imaging study in bipolar disorder to date—a multi-center effort conducted by the ENIGMA bipolar disorder working group—found *no* decrease in cortical measures over time, but in fact slower thinning of specific cortical measures in some brain areas than controls [21]. However, bipolar disorder patients did show an accelerated enlargement of ventricles compared with healthy controls [21]. While the causes of abnormal brain changes remain undetermined, medication use [23, 24], genetic factors [17], and the occurrence of mood episodes [16–21] have been hypothesized as probable contributing factors.

Not only is the occurrence of manic episodes the hallmark of bipolar disorder, but the number of manic episodes has also been associated with worsening illness severity over time [9–13]. The aim of this narrative review was therefore to advance our understanding of the consequences manic episodes might have on neuroanatomical structures. We review and summarize longitudinal structural magnetic resonance imaging studies that relate imaging outcomes to manic episodes. We first provide a brief overview of longitudinal case-control studies in bipolar disorder, then discuss factors that might contribute to structural brain changes, and subsequently, we provide a more detailed outline of studies reporting mania-related structural changes. Based on the reviewed studies, we finally propose a model describing the pathogenesis of mania. Finally, we discuss limitations and suggestions for future research.

## Selection of studies

Since 2011 and up to December 10th 2022, we regularly searched PubMed (NLM) using the following keywords: longitudinal, MRI, structural, neuroimaging, magnetic resonance imaging, mania, manic episodes, mood episodes, bipolar disorder, brain morphology, brain changes, cortical thickness, cortical volume, cortical surface area, subcortical volume, gray matter. Two of the authors (C.A. and L.K.) checked all hits for relevance. We did not rate risk of bias or quality of evidence as this was narrative review. No statistical analyses were performed. We considered only original studies in English and selected 36 studies relevant for structural brain changes in bipolar disorder. Table 1 lists the 7 studies on longitudinal structural brain changes in relation to mania, which was the main focus of this review.

**Table 1:** Overview of the main studies reviewed that related structural longitudinal brain imaging outcomes to manic episodes.

Reference	Diagnosis and sample characteristics	Sample size	Follow-up time	Method; MRI metric	Main result	Brain area affected
Moorhead et al., 2007	Bipolar type 1 (male and female adults).	20	4 years (two timepoints)	VBM/TBM (region of interest); gray matter density	Number of manic/hypomanic episodes during follow-up correlated with gray matter loss in cerebellar and temporal lobe.	Temporal lobe (including hippocampus and fusiform)
Gogtay, 2007	Heterogenous clinical picture with/without first manic episode, healthy controls (male children)	8/9, 18	4–8 years (scanned every two years, incl. scans pre/post first manic episode)	Surface-based; gray matter density	Results suggestive for faster gray matter decreases in children who experienced first manic episode, but not in children that did not experience a manic episode.	Bilateral anterior (and sub genual) cingulate cortex
Abé et al., 2015	Bipolar type 1 with/without manic episodes (male and female adults).	18/21	6 years (two timepoints)	Surface based (region of interest): cortical thickness, surface area, volume	Patients who experienced mania decreased in cortical volume, whereas those without manic episodes did not. Patients who had no manic episodes displayed increases in cortical thickness and decreases in surface area.	Bilateral dlPFC, inferior frontal cortex
Zak et al., 2019	Bipolar type 2 with few/many hypomanic episodes between time points, healthy controls (male and female adults).	12/16, 35	2.4 years (two timepoints)	Surface based (vertex-wise, region of interest, and whole brain)	Patients with few (0–3) hypomanic episodes had greater temporal cortical thinning than patients with more (>3) hypomanic episodes.	left/right temporal cortex
Abé et al., 2020	Bipolar type 1 with/without manic episodes between timepoints, bipolar type 2 with/without hypomanic episodes, healthy controls (male and female adults).	35/21, 8/15, 46	6 years (two timepoints)	Surface based (vertex-wise, whole brain): cortical thickness	Bipolar disorder type 1 patients, who experienced mania, showed greater cortical thinning than controls and patients without manic episodes. The same pattern was observed in bipolar disorder type 2 with respect to hypomania. Cortical change rates did not differ between patients without manic episodes and controls, who displayed normal age-related thinning.	left inferior frontal cortex
Abé et al., 2021	Bipolar type 1, type 2, NOS (male and female adults).	230	0.5–9 years (two timepoints)	Surface based (region of interest, whole brain): cortical thickness, surface area; subcortical volumes	Negative correlation between yearly thickness change rates and the number of manic, hypomanic, and/or mixed episodes between time points. No correlation between the number of mood episodes and surface area or subcortical volumes. Follow-up tests: Patient who experienced manic, hypomanic, and/or mixed episodes showed cortical thinning, while those without showed no changes or increases in cortical thickness. Results remained when excluding patient included in Abé et al. 2015 and 2020, and when controlling for various potential confounders, incl. depressive episodes.	Frontal: left frontal pole, bilateral inferior frontal, right caudal anterior cingulate, left dm/dlPFC. Other brain areas: left lingual, right paracentral, left isthmus, left transverse temporal.
Cahn et al., 2021 (review)	Bipolar type 1 following first manic episode (male and female adults and children).	Sample sizes ranged from 8–41 patients and 17–70 controls	>1 year	Various methods; gray matter volume (cortical or subcortical), cortical thickness, or surface area.	Various findings. Most consistently reported finding was a faster gray matter volume decreases in patients that experienced a first manic episode compared with controls in anterior cingulate cortex.	Anterior cingulate cortex

Sample characteristics, investigated MRI metrics, and main results are summarized.

*dm* dorsomedial, *dlPFC* dorsolateral prefrontal cortex, *MRI* magnetic resonance imaging, *NOS* not otherwise specified, *TBM* tensor-based morphometry, *VBM* voxel-based morphometry.

## Brain morphometric measures

Studies of bipolar disorder have used several brain morphometric measures. The most common are subcortical and cortical volumes. Cortical volume is a function of cortical surface area and cortical thickness, which are two genetically and phenotypically distinct measures [25, 26]. These can be assessed separately by using surface-based measures—provided by, e.g., FreeSurfer [27–30] - to obtain more detailed anatomical information. Cortical surface area is largely determined by the number of cortical columns. Cortical thickness is related to the size, number, and density of cells and dendrites in a cortical column [31]. Cortical thickness therefore serves as a proxy marker of the integrity of the cerebral cortex [1–4, 17, 31]. Here, we considered studies that measured volumes of subcortical and cortical structures, as well as density, thickness, and surface area of cortical structures derived from tensor/voxel-based morphometry or surface-based methods.

## Summary of longitudinal brain changes in bipolar disorder

Even though limited in numbers, studies using a longitudinal case-control design to assess brain changes along the course of bipolar disorder suggest that noticeable structural brain changes in bipolar disorder do occur over time. Several studies report abnormal changes in the prefrontal and temporal cortices as well as in subcortical structures, in particular amygdala [13, 14, 16–19, 21, 22]. For example, in a 6-year follow-up study using vertex-analyses [17], bipolar disorder patients showed an increase of cortical thickness in visual/somatosensory brain areas, whereas healthy controls showed an expected age-related thinning. Differences were observed in the bilateral medial occipital cortex (including pericalcarine and cuneus cortex), bilateral central sulcus, posterior cingulate cortex, and left anterior insula. However, bipolar patients showed a faster cortical thinning than controls in the middle temporal cortex.

More recently, a large multi-center EMIGMA study, including 307 bipolar disorder patients and 925 controls from 14 international sites, investigated yearly change rates of subcortical volumes, regional cortical thickness, and surface area [21]. The thickness change rates of the right fusiform gyrus and right parahippocampal regions differed significantly between bipolar patients and controls: The thickness decreased over time in healthy controls, whereas patients showed less or no decline. No significant difference in surface area or subcortical volumes were noted. The study’s most significant and robust finding was faster ventricular enlargement in bipolar patients than in controls. Interestingly, ventricular change rates correlated negatively with subcortical volume change rates, indicating that bipolar patients with greater ventricle enlargement also had greater subcortical decline over time. While the study populations differed across individual centers (e.g., sample size varied, some were medical trial populations, some patients were followed after their first manic episode), this multicenter study covered a wide range of follow-up periods and leave-one-site-out analyses validated that the reported results were robust. This is the largest longitudinal brain imaging study in bipolar disorder yet performed, and therefore the most relevant for the present review.

In summary, longitudinal brain imaging studies suggest that bipolar disorder in general is associated with deviant brain changes. However, both aberrant decreases and increases in morphometric measures have been reported and it is uncertain if bipolar disorder per se adversely affects the brain.

## Factors contributing to brain changes

The mechanisms underlying observed brain changes in bipolar disorder remain to be elucidated. We outline below some of the most relevant factors that have been suggested to be of importance.

### Pharmacological treatments

Pharmacological treatments of bipolar disorder have diverse effects on brain structure [32]. Lithium—the prototypical mood stabilizer—has been ascribed neurotrophic and neuroprotective properties [24, 33–36]. Lithium use has been associated with increased thickness of the prefrontal and medial occipital cortex [17, 37, 38]. However, such effects need to be interpreted with caution, since lithium itself has an effect on the MR signal which may affect the measured outcomes [39]. Many patients with bipolar disorder are treated with antipsychotic drugs during acute manic episodes, and some patients continue with the antipsychotic medication during the maintenance phase [40]. Gray matter decrease has been associated with antipsychotic drug use [41] - including newer second-generation drugs albeit to a lesser degree [42]. However, findings are mixed with some reviews [43, 44] suggesting that the exact effect of antipsychotic medication on gray matter volumes is still unclear. These inconsistencies are partially due to results being derived from cross-sectional studies, which do not allow conclusions on brain changes over time [45].

### Genetic factors

Bipolar disorder is a highly heritable disorder. Interestingly, cross-sectional studies have found structural differences similar to those in bipolar disorder in unaffected relatives to patients with bipolar disorder [46–49]. Moreover, higher polygenic liability for bipolar disorder and schizophrenia, as indexed by polygenic risk scores, have been associated with thinner ventromedial prefrontal cortex [50]. With respect to specific genetic variants, a recent systematic review of genetic neuroimaging findings from cross-sectional studies concluded that the most consistent finding is the influence of the *CACNA1C* rs1006737 polymorphism on cortical thickness [51], albeit that most studies remain to be replicated.

A longitudinal study found a positive correlation between polygenic risk for bipolar disorder and thickness of medial occipital cortex and central sulcus [17]. This may indicate that genetic factors modulate the risk of disorder-related brain changes over time. However, a longitudinal twin-study reported that the liability to bipolar disorder was not associated with structural brain changes over time [52], whereas a more recent narrative review suggests that heritable changes are primarily confined to white matter [53]. Taken together, longitudinal imaging-genetics studies in bipolar disorder are scarce and show mixed results. Some findings suggest genetic contributions to brain gray matter structure, and possibly a genetic modulation of longitudinal changes.

### Comorbid conditions

Comorbid medical conditions such as substance use disorders, cardiovascular diseases, or obesity may confound observed effects of manic episodes on brain changes. The life-time prevalence of substance use—known to be associated with brain abnormalities [54] and longitudinal brain changes [55, 56] - is high in bipolar disorder, especially during manic episodes. Substance abuse can also trigger manic episodes and affect outcomes and recurrence rates [57, 58]. The risk of adverse cardiovascular events (e.g., stroke) that can adversely affect brain structure [59–61] is higher in bipolar disorder than in the general population, also at younger ages [62]. Bipolar disorder patients are furthermore at increased risk for being overweight or obese, and have elevated risk for metabolic syndrome [63, 64]. In fact, recent studies suggest that comorbid obesity could explain why neurostructural alterations are more pronounced in some individuals with bipolar disorder [65]. Obesity is also a risk factor for accelerated brain ageing in first-episode psychosis patients [66].

Differentiating between bipolar disorder and schizophrenia sometimes poses challenges. Even though these conditions are mutually exclusive according to diagnostic systems, patients might in fact have a lifetime history of both disorders. Psychotic symptoms as seen in schizophrenia are also most commonly observed during manic episodes and, consistent with overlapping symptoms [67], bipolar disorder and schizophrenia share around half of their genetic liability [68, 69]. Schizophrenia is known to be associated with progressive gray matter loss confined to fronto-temporal cortical regions [70]. A greater diagnostic challenge presents the dissociation between bipolar disorder (with psychotic symptoms) and schizoaffective disorder bipolar type (featuring manic episodes) [71]. A recent study aimed at discriminating between schizophrenia, schizoaffective disorder, and psychotic bipolar disorder applying a multimodal brain imaging approach and revealing activity of the salience network as discriminative along the psychosis spectrum [72]. Comparing gray matter volume among these three disorders, schizoaffective disorder displayed on the one hand similar but less extensive gray matter reductions than schizophrenia but on the other hand more gray matter reductions compared to bipolar disorder [73].

These are just to name a few comorbid conditions that are important to consider when investigating longitudinal brain changes in bipolar disorder.

### Accelerated aging

A recent systematic review and meta-analysis found accelerated brain aging in bipolar disorder that was more pronounced in older patients, suggesting the possibility of a cumulative effect of the disease burden [74]. This may also explain the clinical association between bipolar disorder and behavioral variant of frontotemporal dementia [75, 76], as well as the suggested protective effect of lithium against dementia [77]. A recent investigation into the neurobiology underlying age-related brain changes suggest that cortical thinning is associated with inter-regional expression of genes specific to CA1 pyramidal cells, astrocytes, and microglia during both development and aging [78]. In line with this, a study of 1547 bipolar disorder patients showed that cortical thinning is associated with gene expression profile of CA1 pyramidal cells and microglia, and with genes involved in axon guidance during neurodevelopment, synaptic activity, and neuroplasticity [79].

### Manic episodes and neuroinflammatory processes

While depressive episodes [19] may also affect brain structure, several lines of evidence suggest that manic episodes are detrimental for the integrity of cortical structure. Mechanisms underlying pathological gray matter loss may include increased neurodegeneration, neurotoxic susceptibility, neuronal apoptosis, and altered neuroplasticity caused by neuroinflammatory processes and/or oxidative stress during mood episodes [14, 16, 80]. Mania has been related to biochemical changes that may have adverse effects on brain structure and function [81]. For example, stress-induced elevation of cortisol and adrenocorticotrophic hormone has been suggested to play a role during manic episodes [82]. One study found higher cerebrospinal fluid levels of neurofilament light chain—indicative of axonal damage—in bipolar disorder compared with healthy controls [83], and a recent longitudinal study showed increased concentrations of an oxidative stress marker in cerebrospinal fluid following mania [84]. With respect to neuroinflammation, higher cerebrospinal fluid concentrations of interleukin 8 [85] and interleukin-1ß [86] was found in bipolar disorder patients compared with controls. Manic episodes have also been associated with immunological activation of microglia [87], with perisynaptic release of proinflammatory cytokines such as interleukin-1ß, interleukin-2, and tumor necrosis factor [11, 88]. Abnormal synaptic plasticity involving altered secretion of neurotrophic factors could be at play during mania, as a recent study found lower levels of secretogranin II—a neuroprotective compound that reflects secretion of neurotrophins—in patients with bipolar disorder type 1 (but not type 2) compared with controls [89].

## Brain changes associated with manic episodes

Early cross-sectional studies suggested that the number of manic episodes correlated negatively with gray matter volume in dorsolateral prefrontal cortex (dlPFC) [90] and inferior frontal cortex [91]. However, it was not possible to determine if these correlations reflect temporal relationships between brain changes and manic episodes, or whether premorbid trait abnormalities increased the risk for manic episodes. Longitudinal studies can address this question. Below we summarize the findings from longitudinal studies that related structural brain imaging outcomes to manic episodes. In Table 1, we present the characteristics of the studies discussed.

In 2007, Moorhead and coworkers [18] performed the first structural longitudinal brain imaging studies in bipolar disorder type 1 using voxel-based morphometry to measure gray matter density. This 4-year follow-up study of 20 patients reported that the number of manic/hypomanic episodes correlated with gray matter loss. Although the study was small and limited the investigation to temporal cortex where case-control differences had been observed, it was the first indication that manic episodes may be related to gray matter changes.

A decade later, Abé and colleagues examined 13 bipolar I disorder patients who had experienced manic episodes during a mean follow-up time of six years along with 18 patients who had not [16]. The study used a region of interest approach and investigated cortical volume, thickness, and surface area. Patients who had experienced manic episodes showed faster volume decrease in the dlPFC (comprising the superior frontal, caudal middle frontal, and rostral middle frontal cortex) and the inferior frontal cortex (including pars opercularis, pars triangularis and pars orbitalis). Intriguingly, patients who did not experience manic episodes showed no volume changes, but increases in thickness and decreases in surface area were indicated. The mania-related changes in dlPFC volume were driven by changes in rostral middle frontal cortex volume. Sensitivity analyses showed that the results remained when patients with more than one manic episode were excluded, suggesting that even a single manic episode may adversely affect cortical volume. However, no healthy controls were included in this study, which precluded conclusions about how the changes related to normal ageing.

In 2020, the study by Abé and coworkers [16] was extended to include both bipolar type 1 (including some patients from the previous study), bipolar type 2, and healthy controls [17]. This study used a validated longitudinal image processing pipeline and more advanced data analyses, e.g., linear mixed effects modeling, as well as a more fine-grained vertex-wise anatomical analysis. After the follow-up period of 6 years, similar effects of manic, hypomanic and/or mixed episodes on cortical thickness were observed in left inferior frontal cortex. The study also indicated that the mania-related changes reflected accelerated decline compared with controls. The findings remained when the previously investigated patients were excluded [16], and similar results were obtained after adjusting for depressive episodes between timepoints. Finally, change rates in inferior frontal cortex correlated positively with change rates in middle temporal cortex indicating a potential mania-related decline in other brain areas.

**Fig. 1:**
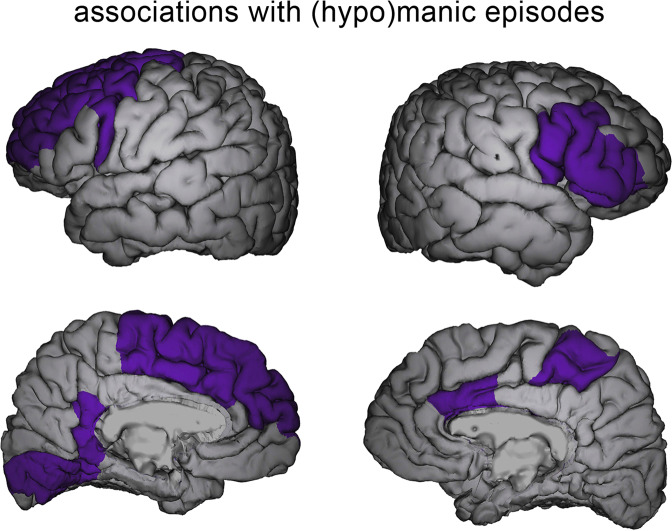
Associations between longitudinal cortical thickness changes and (hypo)manic episodes. Figure obtained from Abé et al. [21] showing anatomical locations of brain regions in which negative correlations between thickness change rates and the number of (hypo)manic episodes between imaging timepoints were observed in the ENIGMA-BD study [21]. The brain areas identified in the ENIGMA-BD study largely cover regions identified in this review (see Table 1 for details).

In 2022, the largest longitudinal neuroimaging study in bipolar disorder to date was published [21]. This international multi-center study, conducted within the ENIGMA consortium, investigated yearly change rates of 11 subcortical regions and 64 cortical brain regions covering the whole brain. Changes in subcortical volume, as well as cortical thickness and surface area were quantified. The study found negative correlations between the number of manic episodes between imaging time points and cortical thickness change in frontal pole and the lingual gyrus of the medial occipital cortex. When considering the number of all manic, hypomanic, and/or mixed mood episodes, negative correlations were observed with the medial occipital cortex and in widespread manner over frontal cortex—including dlPFC and inferior frontal cortex—and anterior cingulate cortex (Fig. 1). The results remained after adjusting for the number of depressive episodes between timepoints, after excluding the cohort previously reported by Abé and colleagues [17], as well as after excluding first-episode mania cohorts. Further analyses suggested that more frequent manic episodes were associated with faster cortical thinning, and that patients with no manic episodes showed no change or increased cortical thickness depending on brain region.

Since most studies reviewed above included patients that had had bipolar disorder for many years, they had received treatment and sometimes experienced several manic episodes at the first scanning. To disentangle effects of mood episodes per se from those of illness duration and medication, it is valuable to investigate patients in proximity to the onset of their first manic episode. In 2007, Gogtay and colleagues conducted a pre-post onset longitudinal imaging study in pediatric bipolar disorder [92]. The authors suggested that, after onset of bipolar I disorder, i.e., after the first manic episode, children may show faster gray matter decreases bilaterally in the anterior (and sub-genual) cingulate cortex than those that did not develop bipolar disorder type 1. Note that the investigated samples were small (*n* = 8–9 per group) and the results were only suggestive and not statistically significant in direct group comparisons. Also, the children were ‘multi-dimensionally impaired’, i.e., they presented with a heterogenous clinical picture, comprising emotion dysregulation, attention deficit hyperactivity disorder, developmental disorders, and psychosis. Most recently, van Rheenen et al. revealed that first episode of mania in adolescent and young adults (age 15–25) did not impact cortical thickness or gyrification but increased surface area was found in inferior and middle prefrontal and occipitoparietal cortices [93].

A recent review on longitudinal gray matter changes following the first mania in bipolar disorder type 1 included 15 studies (the study by Gogtay et al. was not included) and concluded that the most replicated finding was a decrease in anterior cingulate cortical volume following first mania [22]. Although conclusions on regional specificity may be difficult given the inconsistency of findings and methodology applied in the individual studies, this report suggests changes in prefrontal brain structural following the first manic episode in bipolar disorder.

Hypomania denotes a milder form of mania [94]. Patients with bipolar disorder type 2 experience recurrent depressive and hypomanic episodes, but, by definition, no manic episodes. In the study by Abé and colleagues, the pattern of mania-related inferior frontal cortical thinning in bipolar disorder type 2 was also observed for hypomanic episodes in bipolar type 2 patients [17]. In contrast, however, Zak and coworkers reported that bipolar disorder type 2 patients with few (0–3) hypomanic episodes between baseline and follow-up scan had greater temporal cortical thinning than patients with many (>3) hypomanic episodes [19]. However, no correlations between the actual number of mood episodes were observed and the dichotomization of continuous variables performed in this study hamper the interpretation [95].

Taken together, the most consistent finding is that manic episodes relate to brain changes in prefrontal brain areas. Changes in temporal regions have been reported as well, but less consistently.

## Prefrontal cortical integrity trajectory in relation to the occurrence of manic episodes

**Fig. 2:**
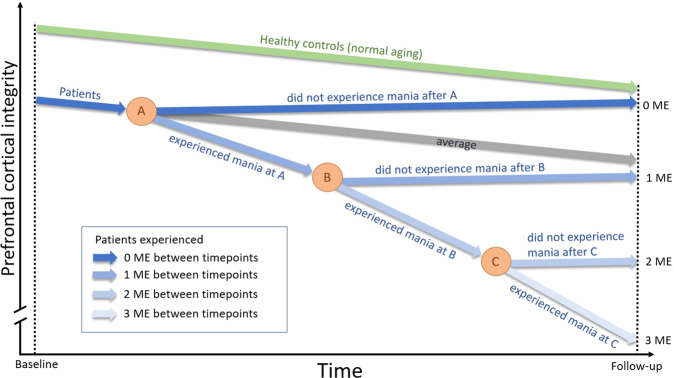
Cortical integrity trajectory model. This simplified scheme illustrates structural gray matter changes in relation to the occurrence of manic episodes in bipolar disorder type 1. This model is based on results of the reviewed longitudinal studies depicting changes in prefrontal cortical integrity, the area most consistently reported, as representative example. Structural integrity is defined by the various structural brain imaging outcomes investigated in these studies (e.g., cortical thickness). Three events in time (A, B, C) between baseline and follow-up scans are indicated, at which a manic episode may or may not have occurred. Healthy control trajectory is shown in green, patient trajectories in blue. Patient average trajectory (gray) is based on general longitudinal findings showing no case-control differences with respect to change rates. Different shades of blue show example trajectories for patients who experienced none, one, two, or three manic episodes (ME) between imaging time points. Arbitrary units are used to express approximate trends. Additional notes: The illustration assumes that patients and controls are age matched at baseline. Although age-related slopes are not necessarily linear and may depend on age and follow-up period, linear slopes were illustrated for simplicity. Further, the average trajectory (gray arrow; same slope as controls) could potentially result from counterbalancing sub-group trajectories, where one group declines faster than controls and one show increased gray matter. This could potentially be the case if some patients experienced mania shortly before baseline imaging time point.

Based on the studies reviewed, a picture emerges where periods of inter-episodic euthymia are associated with no structural change or even an increase in gray matter, whereas manic episodes are associated with gray matter decrease predominantly in the prefrontal cortex. Hence, longitudinal studies suggest that the structural prefrontal cortical integrity is unaffected or improved in the absence of manic episodes: On average, patients follow a similar trajectory as healthy individuals and the slope is similar to that of normal age-related cortical decline. It is in relation to manic episodes that the prefrontal cortical integrity deteriorates. Figure 2 illustrates proposed trajectories of how prefrontal cortical integrity may change over time in relation to the occurrence of manic episodes.

However, cross-sectional studies report lower frontal cortical volume and thickness in bipolar disorder [2–4]. These aberrancies may be consequences of manic episodes accumulated before first imaging timepoint. They may also reflect static/premorbid conditions associated with the disorder that are unrelated to previous manic episodes. Therefore, and regardless of the origin of such baseline abnormalities, the proposed trajectory model shows lower cortical integrity in bipolar disorder at baseline (Fig. 2).

Available data suggest that when bipolar patients experience manic episodes, structural brain metrics decrease faster not only in comparison with healthy controls but also compared with patients that remain well (Fig. 2, line labeled ‘experienced mania at A’). Since the degree of decline seems to correlate with the number of manic episodes between imaging timepoints [21], each additional manic episode presumably add to the abnormal gray matter decline, and the change rate might even accelerate (Fig. 2, line labeled ‘experienced mania at B/C’). Importantly, however, we also propose that patients who remain well may show structural recovery if manic episodes can be prevented (related or unrelated to treatment). This proposition rests on some studies reporting that structural brain measures do not change or increase in patients that did not experience manic episodes between timepoints (Fig. 2, lines labeled ‘did not experience mania after A/B/C’). It should be noted that size increases of cortical structures do not necessarily reflect beneficial effects. They may also be explained by e.g., neuroinflammatory processes, previously suggested to occur in bipolar disorder [96]. However, as stated above, the neurobiological mechanisms behind the observed structural changes remain to be clarified.

## Limitations of previous studies and suggestions for future research

Even though longitudinal studies can distinguish static abnormalities from dynamic changes, causal links to associated factors cannot be established. It thus remains unclear whether structural changes cause manic episodes or vice versa. It is also unknown if mania induces brain changes directly, e.g., through biochemical mechanisms as discussed above, or whether a third unrelated factor causes both structural decline and mania. To answer such questions and to validate the proposed model, we need larger datasets designed to specifically study brain trajectories with a fine-grained time resolution. Of great interest would be studies covering the period around the first manic episode, preferably in a pre- and post-first mania design similar to that used in [92].

Although this review focuses on the association of structural brain changes and mania, bipolar patients also experience episodes of depression, often severe and long lasting, which might also be associated with brain changes. In addition, medication effects also need to be disentangled from mania-related effects. For example, although challenged and still inconclusive [43, 44], antipsychotic drug use has been associated with gray matter decrease [41]. Moreover, lithium use has been associated with gray matter volume increases [33, 36, 37] and has been attributed neuroprotective effects [34, 35], which might explain the observation that some patients who remain well show no cortical decline or even structural recovery. Since medication effects may be confounded by indication, these are better addressed in interventional experimental study designs, such as randomized controlled trials. A recent study examining brain structure networks investigated individuals with a manic episode over the course of treatment with quetiapine or lithium: Unmedicated manic patients featured global and nodal network deviances compared with controls, which, however, normalized with treatment [97].

Another important factor to consider is that bipolar disorder is highly heritable. Thus, genetic effects may play a role in structural brain trajectories over time. For example, Abé and colleagues found relationships between brain changes and polygenic risk scores for bipolar disorder and schizophrenia that warrant further investigation [17]. Finally, comorbidity with other medical conditions may be crucial to consider as they may affect brain trajectories over time.

Apart from disentangling mania effects from other factors contributing to gray matter changes, it is important to investigate if mania-related brain changes translate to changes in symptomatology, as well as social and cognitive functioning. Meta-analyses suggest that executive functions are impaired in bipolar patients [98], but it is less clear how such cognitive measures change over time and how they relate to structural brain changes. There are longitudinal studies indicating that the number of manic episodes relates to changes in cognitive function [99], but the findings in other studies are mixed [100].

Although this review focuses on structural brain imaging findings, other imaging modalities like functional imaging (fMRI) and diffusion tensor imaging (DTI) also have the potential to advance our understanding of mania-related brain alterations in BD [101–104]. To date, however, longitudinal studies of mania-related changes are scarce. Hence, future longitudinal studies using fMRI and/or DTI are warranted to further our understanding of how manic episodes cause longitudinal functional and white matter integrity changes, respectively.

## Conclusion

In this narrative review, we summarized results from longitudinal studies in bipolar disorder that relate structural brain imaging outcomes to manic episodes. The results suggest that mania is associated with brain changes, i.e., decreases in gray matter metrics, most consistently reported in the prefrontal cortex. Evidence also suggests increases in brain metrics if manic episodes do not occur. The latter potentially reflects structural improvement mechanisms and emphasizes the importance of preventing manic episodes. Finally, to pinpoint targets for improving outcomes, future studies should attempt to disentangle the effects of mania from other factors associated with brain changes.
